# May-Thurner Syndrome and Patent Foramen Ovale: A Rare Etiology of Cryptogenic Stroke

**DOI:** 10.7759/cureus.5468

**Published:** 2019-08-23

**Authors:** Benjamin J Phelps, Otto Boutin, Urvesh Patel, Christopher King

**Affiliations:** 1 Internal Medicine, Palmetto General Hospital, Hialeah, USA; 2 Critical Care, Palmetto General Hospital, Hialeah, USA; 3 Critical Care and Internal Medicine, Palmetto General Hospital, Hialeah, USA

**Keywords:** may-thurner, cryptogenic stroke, cerebrovascular accident (cva), patent foramen ovale, pfo closure, may-thurner syndrome, intravascular ultrasound

## Abstract

Patients who present with stroke or transient ischemic attacks (TIA) in the setting of patent foramen ovale (PFO) mandate investigation of the lower extremities and pelvis in order to determine a possible source of thromboembolic disease. Imaging studies including Doppler ultrasound of the extremities may not be sufficient to diagnose the presence of anatomic variants that predispose patients to thrombus formation. May-Thurner syndrome (MTS) is characterized by extrinsic compression of the common iliac veins or inferior vena cava which leads to chronic physiologic changes within the vasculature. This condition increases risk of venous occlusion, diminution of venous flow, and most significantly, formation of thrombi. In this case report, we present a young Hispanic female diagnosed with ischemic cerebral vascular accident (CVA) secondary to thromboembolism in the setting of May-Thurner syndrome and a PFO, a rare etiology of cryptogenic CVA.

## Introduction

May-Thurner syndrome (MTS) is characterized by extrinsic compression of the common iliac veins or inferior vena cava which leads to physiologic changes within the vasculature. Patients diagnosed with this condition are predisposed to venostatic disease secondary to varying degrees of occlusion with or without thrombus formation. The pathophysiology of MTS involves extrinsic venous compression by the arterial system onto bony structures causing a focal stenosis of the compromised vessel [1]. These physiologic changes potentiate thrombus formation and obstruct outflow from the ipsilateral lower extremity. The overall incidence of this condition is unknown. Clinical features of MTS, when symptomatic, include extremity swelling, claudication, and chronic skin changes associated with venous insufficiency. A rare complication of MTS includes cryptogenic stroke in patients with patent foramen ovale (PFO) [2]. This case report describes a young Hispanic female who presented with new onset neurological deficits and was diagnosed with a thromboembolic stroke as a result of PFO in the setting of MTS.

## Case presentation

A 45-year-old Hispanic female presented to our community hospital with sudden onset of expressive aphasia, dysarthria and right upper extremity weakness. This patient was last known well one hour prior to arrival. Head computed tomography (CT) demonstrated a subacute hypodensity in the left cerebellum. Perfusion CT imaging demonstrated a deficit on mean transit time concerning for watershed type stroke versus a distal left middle cerebral artery occlusion.

The patient has a past medical history of hyperlipidemia and chronic anemia status post gastric bypass surgery several years prior to presentation. She denied alcohol, tobacco, or illicit drug use. She denied family history of stroke, heart conditions, or coagulopathies. Her home medications included atorvastatin 40 mg PO daily and cyanocobalamin 1000 mcg PO daily.

**Figure 1:**
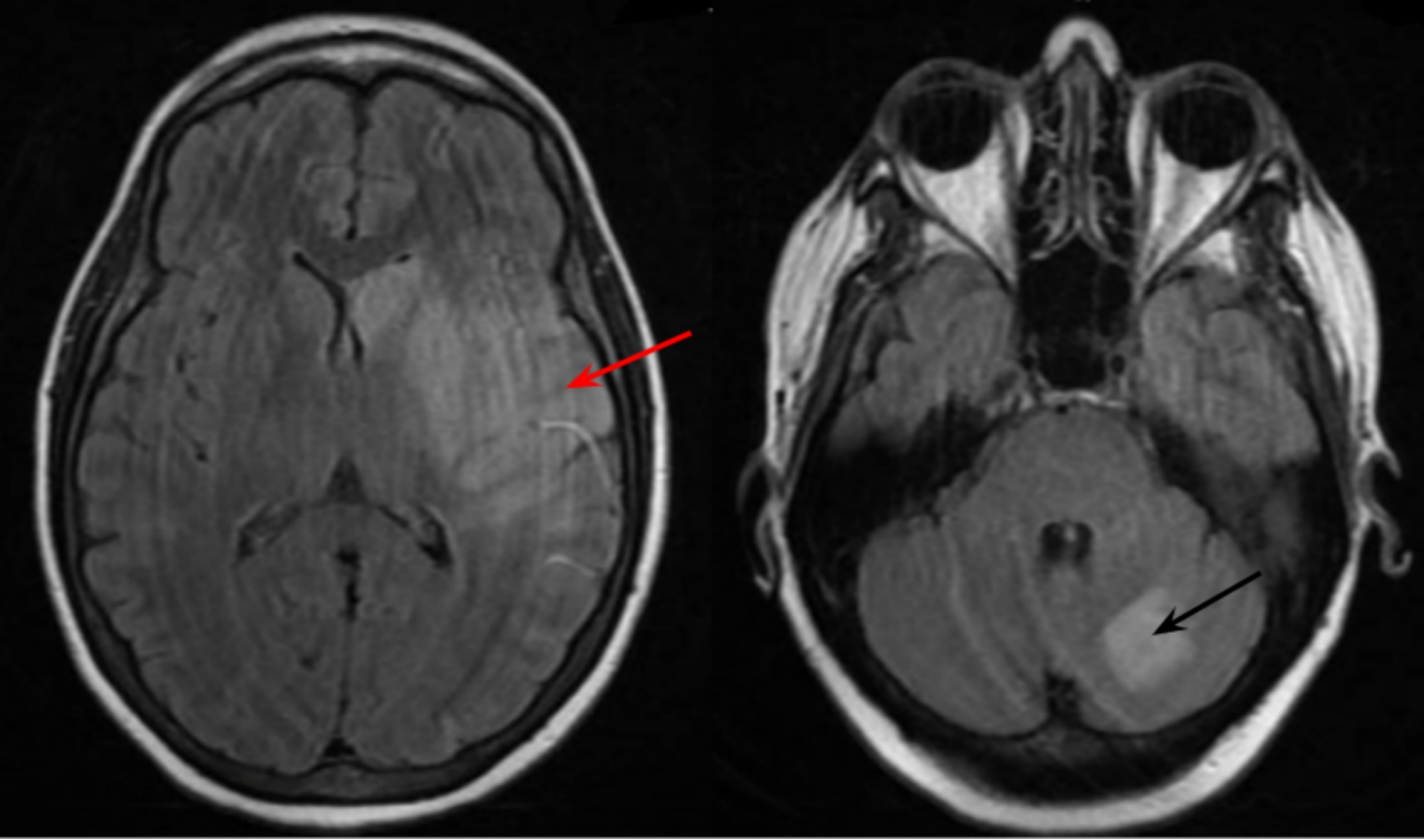
Magnetic resonance imaging of the brain demonstrating multiple large and small infarcts of the left parietal lobe and basal ganglia (red arrow) with an older infarct of the left cerebellum (black arrow) consistent with thromboembolic disease.

On the first day of admission, the patient underwent emergent neurointervention with cerebral angiogram and mechanical thrombectomy of the left middle cerebral artery secondary to significant findings on CT cerebral perfusion imaging. Following intervention, the patient was globally aphasic with gross right upper and lower extremity weakness but able to follow simple commands. Magnetic resonance imaging (MRI) of the brain demonstrated multiple large and small infarcts of the left parietal lobe and left basal ganglia, with an older infarct of the left cerebellum (Figure 1). Given the distribution of multiple areas of infarction on MRI, it was suspected that the source of ischemic stroke was thromboembolic in nature.

**Figure 2:**
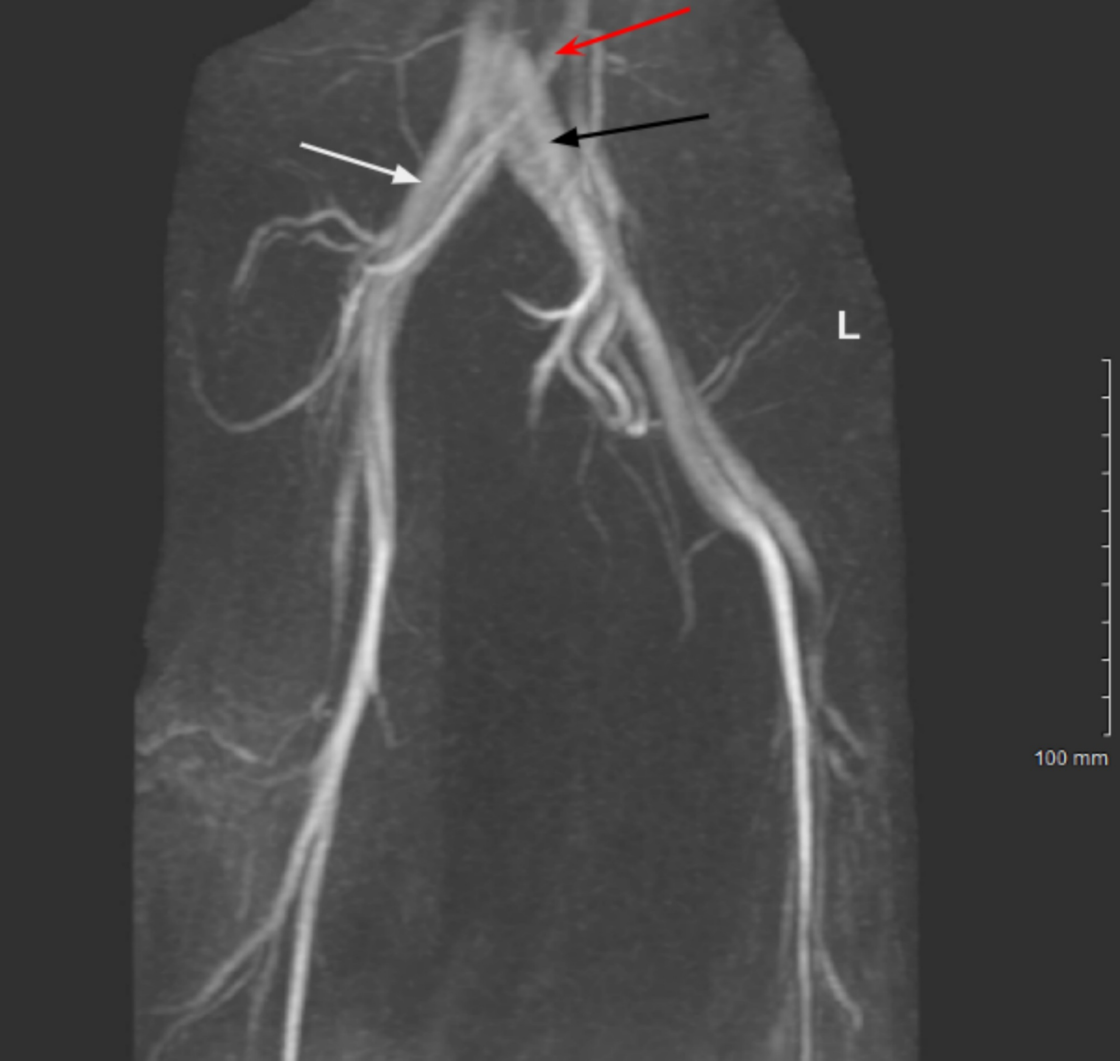
Magnetic resonance venography of the pelvic vasculature demonstrating significant narrowing of the left common iliac vein (black arrow) at the level of the right common iliac artery (red arrow). The right common iliac vein is of normal course and caliber (white arrow).

Transesophageal echocardiogram demonstrated a positive agitated saline test confirming the presence of a patent foramen ovale. CT angiogram of the chest demonstrated minimal minute filling defects of the right inferior pulmonary arteries consistent with small pulmonary emboli. Doppler ultrasound studies of bilateral upper and lower extremities were negative for thrombus. Hypercoagulable workup including testing for antiphospholipid antibodies, factor V Leiden, protein C, protein S, antithrombin III activity, prothrombin gene mutation, and homocysteine levels were unremarkable. Further investigation into thrombogenic source was warranted and magnetic resonance angiography (MRA) and magnetic resonance venography (MRV) of the pelvis were completed. Three-dimensional (3D) reconstruction of the imaging studies revealed a focal narrowing of the left common iliac vein at the level of crossing of the right common iliac artery (Figure 2). To date, the patient had denied symptoms of lower extremity edema or claudication and had never demonstrated physical findings of chronic venous insufficiency. The patient underwent intravascular ultrasound (IVUS) and fluoroscopic-guided iliocaval venography which demonstrated a 52% stenosis of the distal portion of the left common iliac vein at the level of the right common iliac artery compatible with May-Thurner pathology. After discussion and review of the imaging with the patient, she elected to undergo minimally invasive intravascular stenting of the left common iliac vein.

Given her history of multiple cerebral infarctions and recent diagnosis of May-Thurner syndrome, the patient was determined to be at risk for recurrent cerebral vascular accident (CVA) and underwent closure of the PFO. Her Risk of Paradoxical Embolism (RoPE) score was calculated to be 7, demonstrating a 72% probability the stroke was due to a pathologic PFO and a 6% chance of recurrent CVA [3]. The patient received supportive care, daily physical therapy, and speech therapy for the duration of her hospital course. The patient was started on dual antiplatelet therapy and full dose anticoagulation prior to discharge to a rehabilitation facility. With therapy, the patient had improvement of her right hemiplegia and aphasia.

## Discussion

This patient was determined to have a PFO with transesophageal echocardiogram (TEE) on routine investigation following a CVA. PFO often are undiagnosed as this type of congenital lesion is relatively common in the normal population and may remain asymptomatic. Hagen et al. found PFO in 25 to 30 percent of subjects in community-based study at autopsy using TEE [4]. Meissner et al. evaluated over 500 patients over the age of 45 for potential risk factors of stroke using TEE and revealed a prevalence of 26% [5]. PFO defects are not always pathologic as some may be found coincidentally in patients presenting with stroke who have vascular risk factors such as smoking or diabetes. The RoPE study is a meta-analysis which reviewed over 3000 patients with cryptogenic stroke and discovered the likelihood that stroke was PFO-attributable correlated with the absence of vascular risk factors and the presence of cortical cryptogenic infarcts on imaging. Using this information, the investigators derived a predictive model for statistical relatedness of PFO to stroke. The RoPE score uses risk factors including hypertension, history of diabetes, smoking history, prior transient ischemic attacks or strokes, age, and findings on imaging to estimate the probability of a pathologic PFO in patients with cryptogenic stroke. The score also predicts the risk of stroke recurrence at two years based on the same risk factors [3,6]. In our patient, her RoPE score was calculated to be 7, which demonstrated the PFO-attributable fraction to be 71.1%. Given her young age and imaging findings, we suspected her PFO to be pathologic and further investigation into a source of the thromboembolic phenomenon was warranted. The presence of pulmonary embolism in this patient added to the suspicion of paradoxical emboli causing stroke and further imaging of the pelvis was conducted.

May-Thurner syndrome was identified in this patient with MRV of the pelvis and further characterized by contrast venogram with IVUS. MTS is an important etiology of thrombotic disease that warrants consideration in young patients suffering from cryptogenic stroke. The pathophysiology behind MTS is relatively unknown. Risk factors for MTS include female gender, scoliosis, dehydration, and hypercoagulable disorders. Patients with MTS can present with acute or chronic unilateral lower extremity pain or swelling, as well as recurrent ulcers, varicose veins, and skin pigmentation changes of the lower extremity [7]. The prevalence of MTS is also unclear as patients suffering with pelvic venous compression, even those with significant stenosis, are not necessarily symptomatic [1,8]. This is likely attributable to formation of various collateral pathways for venous return. A study by Kibbe et al., in which 50 patients underwent CT imaging of the abdomen and pelvis secondary to abdominal pain, demonstrated the prevalence of hemodynamically significant lesions, defined as greater than 50% stenosis of the affected vessel, was 25% without lower extremity symptoms [9]. Tests commonly used to assess the structure and function of the venous system of the lower extremities include Doppler ultrasound, MRV, and contrast venography. Our patient underwent Doppler ultrasound of the lower extremities and pelvis during her initial evaluation, however this modality failed to reveal the pathology within the pelvic vasculature. In the evaluation and assessment of pelvic and intra-abdominal veins, Doppler ultrasound is less accurate and highly operator-dependent [10]. The gold standard for diagnosis of MTS has been contrast venography [10], however more accurate methods are currently being utilized. IVUS has been shown to be superior to contrast venogram in the assessment of the degree and extension of iliofemoral occlusive lesions [11]. Our patient underwent contrast venography with IVUS to evaluate severity of disease and provide a therapeutic modality with intravascular stenting of the affected vessel. Intravascular ultrasound is an invaluable imaging modality for establishing a diagnosis of MTS as the sensitivity and specificity of IVUS for venous stenosis exceeds 98% [12].

In one retrospective study, 470 patients that underwent PFO closure secondary to cryptogenic stroke were reviewed. They found that 30 patients (6.3%) had features consistent with MTS on imaging studies. Most of these patients were female, ages 32-54. The study found that the majority of the patients were asymptomatic from venous obstructive symptoms of the lower extremity. They suggest in the context of a previous embolic event, asymptomatic MTS may be an important clinical association and screening with pelvic MRV may be warranted [2].

Although frequently overlooked, MTS is important pathologic condition associated with cryptogenic stroke and PFO. Further diagnostic imaging to investigate pelvic vascular anatomy utilizing MRV and IVUS should be considered in the context of embolic disease and CVA.

## Conclusions

Cryptogenic stroke as a result of PFO in the setting of paradoxical embolism is an established etiology of neurologic disease. May-Thurner syndrome often goes overlooked as a potential source of thromboembolism and further diagnostic imaging using newer modalities such as MRV and intravenous ultrasound to investigate the vascular anatomy of the pelvis should be considered in the context of embolic disease and CVA.
